# Extreme Variability of the Kidney Hilar Architecture: A Radioanatomical Map to Guide Surgical Approaches

**DOI:** 10.3390/diagnostics16101544

**Published:** 2026-05-19

**Authors:** Daniel Gondorf, George Triantafyllou, Ioannis Paschopoulos, Nikolaos-Achilleas Arkoudis, Georgios Velonakis, Panagiotis Kokoropoulos, Nikolaos Arkadopoulos, Maria Piagkou

**Affiliations:** 1 Department of Anatomy, School of Medicine, Faculty of Health Sciences, National and Kapodistrian University of Athens, 11527 Athens, Greece; daniel.gondorf@gmail.com (D.G.); georgerose406@gmail.com (G.T.); johnpascho@gmail.com (I.P.); 2 Research Unit of Radiology and Medical Imaging, National and Kapodistrian University of Athens, 11527 Athens, Greece; nick.arkoudis@gmail.com (N.-A.A.); gvelonakis@med.uoa.gr (G.V.); 3 Second Department of Radiology, General University Hospital “Attikon”, National and Kapodistrian University of Athens, 12462 Athens, Greece; 4 Fourth Department of Surgery, General University Hospital “Attikon”, National and Kapodistrian University of Athens, 12462 Athens, Greece; kokoropoulos@yahoo.gr (P.K.); narkado@hotmail.com (N.A.)

**Keywords:** renal artery, renal vein, kidney hilum, anatomical variation, retroperitoneal surgery

## Abstract

**Background:** Retroperitoneal surgical corridors, particularly in robotic-assisted partial nephrectomy and donor surgery, require precise knowledge of hilar vascular orientation. The typically described Vein–Artery–Pelvis (VAP) anatomy is often assumed, yet its reliability is poorly quantified. Therefore, the purpose of the present study is to provide a comprehensive radioanatomical map of hilar architecture to enhance surgical safety and predictability. **Methods:** Contrast-enhanced computed tomography (CT) scans of the abdomen from 200 patients (104 males and 96 females) were evaluated. The anterior-to-posterior sequence of hilar structures, the frequency of early vascular branching, and the presence of accessory vessels were documented and stratified by sex and laterality. **Results:** The conventional VAP sequence was observed in a minority of cases, occurring in only 32.6% (131 sides). The map identified 37 distinct sequence variants. The most common variants included VAPA (9.0%), AVAP (7.8%), and VAAP (7.0%). Adherence to typical VAP anatomy was significantly lower in males (27.9%) than in females (37.7%). Arterial complexity, characterized by early branching or accessory vessels, was present in 43.2% of sides, with a significantly higher occurrence in males and on the right side. Venous mapping revealed a marked lateral disparity; accessory veins were predominantly right-sided (12.5%), whereas early venous branching was predominantly a left-sided feature (30.0%). **Conclusions:** Renal hilar architecture demonstrates substantial variability, with the classical VAP configuration representing a minority arrangement. These findings highlight the importance of individualized preoperative imaging assessment and may help anticipate anatomical complexity in retroperitoneal surgery.

## 1. Introduction

The renal hilum is a critical anatomical region in retroperitoneal surgery. This gateway serves as the convergence point for the kidney’s primary vascular, urothelial, lymphatic, and neural components. Their spatial configuration and sequence directly influence surgical safety and technical complexity [1]. Contrast-enhanced computed tomography (CT) has emerged as the primary modality for preoperative evaluation of renal vascular anatomy, enabling detailed three-dimensional assessment of hilar relationships [2]. Surgical exposure of the renal hilum is essential in an array of procedures, including radical and partial nephrectomy, pyeloplasty, retroperitoneal lymph node dissection, and kidney procurement for transplantation [3]. In these scenarios, misinterpretation of the vascular sequence or failure to recognize variant vessels may lead to significant hemorrhage, ischemic injury, or the loss of functional renal parenchyma. These risks are especially heightened in minimally invasive and robotic retroperitoneal approaches, where the restricted operative field demands meticulous preoperative anatomical mapping [2].

Renal arterial variants are among the most clinically significant vascular anomalies. Accessory renal arteries (ARAs), defined as additional vessels arising independently from the aorta, are present in approximately 20% of individuals [4]. Early branching renal arteries (ebRAs), wherein division occurs prior to hilar entry, represent a distinct anatomical variant with direct implications for vascular control and segmental perfusion strategies [5]. Likewise, renal venous variants—including early-branching renal veins (ebRVs) and accessory renal veins (ARVs) introduce additional complexity to hilar dissection and venous management [6]. Significantly rarer, other variations in the renal vessels have been described, such as fenestrations [7,8] and retroaortic course [9,10].

Despite the clinical significance of these variations, studies integrating hilar sequence patterns with arterial and venous variant classification in large imaging cohorts—particularly with stratification by sex and laterality—remain limited. Consequently, this study undertakes a large-scale radioanatomical analysis to systematically characterize hilar architecture. We hypothesize that the classical VAP configuration represents a minority pattern and that renal hilar anatomy demonstrates systematic variation influenced by sex and laterality. Additionally, our objective is to develop a systematic radioanatomical framework (categorizing hilar anatomy into simple, intermediate, and complex configurations) to support preoperative anatomical assessment in retroperitoneal renal surgery.

## 2. Materials and Methods

### 2.1. Sample Demographics

The present retrospective study analyzed 200 contrast-enhanced abdominal computed tomography (CT) examinations (400 renal units) obtained from the National and Kapodistrian University of Athens. Ethical approval was granted by the institutional review board (approval number: 1037; date: 28 April 2025). The scans were performed between January 2022 and December 2024. The study cohort included 104 males and 96 females, with a mean age of 63.07 ± 14.3 years (range: 22–91 years).

### 2.2. Inclusion and Exclusion Criteria

Inclusion criteria comprised adult patients (≥18 years) who underwent contrast-enhanced abdominal CT with adequate image quality allowing clear visualization of the renal hilum and associated vascular structures.

Exclusion criteria included patients with prior renal surgery, congenital renal malformations, renal tumors (>4 cm), significant renal atrophy, or severe hydronephrosis, as these conditions can substantially displace or distort the baseline hilar architecture. Additionally, CT scans with poor image quality, motion artifacts, or incomplete visualization of the renal vessels were excluded from the analysis.

### 2.3. Radiological Protocol

All examinations were performed using a multidetector CT scanner (Canon Medical Systems, Otawara, Japan). Imaging was performed using a contrast-enhanced biphasic abdominal CT protocol. Intravenous iodinated contrast medium (370 mg iodine/mL) was administered via a power injector at a rate of approximately 4 mL/s, followed by a saline flush. The arterial phase was initiated using bolus tracking (triggered at 150 HU within the abdominal aorta) with an average delay of 25–30 s, while the portal venous phase was acquired at 70 s. Images were reconstructed with a slice thickness of 1.0 mm and a reconstruction interval of 0.8 mm. Post-processing and image analysis were conducted using Horos software version 3.3.6 (Horos Project, New York, NY, USA). Each dataset was systematically reviewed in axial, coronal, and sagittal planes, with supplementary three-dimensional volume-rendered reconstructions generated to enhance anatomical visualization and confirm vascular relationships.

### 2.4. Methodology

Renal hilar architecture was evaluated using multiplanar reconstructions, with particular emphasis on coronal and sagittal views. Hilar configurations were classified according to the number and sequence of structures entering or exiting the hilum, using the standardized abbreviations vein (V), artery (A), and pelvis (P), along with their respective variants. The anterior-to-posterior sequence of hilar structures was determined at the medial hilar plane—defined as a reproducible vertical reference plane at the level of the medial border of the renal parenchyma, corresponding to the point of vascular entry and exit. The sagittal plane was utilized as the primary reference for recording the sequence. To ensure accuracy in complex cases, findings were cross-referenced with axial and coronal multiplanar reconstructions.

Arterial and venous variations were assessed primarily on coronal reconstructions. Renal arteries were classified as ARAs when supernumerary vessels originated independently from the aorta, or as early-branching renal arteries when a single arterial trunk divided proximal to the hilum. RVs were classified as ARVs when supernumerary veins drained independently into the inferior vena cava, or as ebRVs when multiple venous branches exited the hilum and converged prior to entering the inferior vena cava. These definitions were applied in accordance with previously published criteria.

### 2.5. Statistical Analysis

Statistical analysis was performed using IBM SPSS Statistics for macOS (version 29; IBM Corp., Armonk, NY, USA). Categorical variables were expressed as frequencies and percentages. Side-related paired comparisons were evaluated using the McNemar-Bowker test (an extension of the McNemar test for *k* × *k* tables). To account for potential clustering arising from analyzing two renal units per patient, sex-based comparisons were performed using Generalized Estimating Equations (GEEs) with a binomial distribution, logit link function, and an exchangeable working correlation structure. This method was selected over the chi-square to adjust standard errors and account for intra-patient correlation between the left and right kidneys. All statistical tests were two-sided, and *p*-values < 0.05 were considered statistically significant. Interobserver reliability was assessed using Cohen’s kappa coefficient for categorical variables. Two independent reviewers performed all evaluations, and discrepancies were resolved through consensus with senior authors. A kappa value of 0.60 or greater was interpreted as indicating at least moderate agreement.

## 3. Results

**Figure 1:**
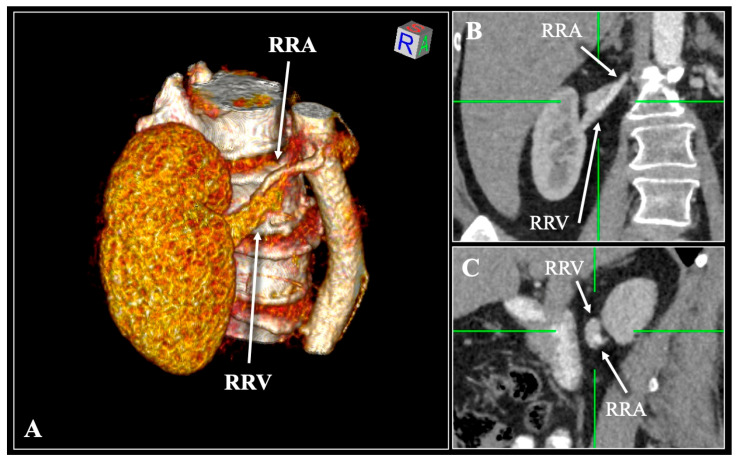
Contrast-enhanced computed tomography (CT) scan of a 63-year-old female patient demonstrating the typical Vein (V)—Artery (A)—Pelvis (P) anatomy. Three-dimensional reconstruction (**A**) and multiplanar reconstruction with coronal (**B**) and sagittal (**C**) slices. A single right renal artery (RRA) and vein (RRV) are entering/exiting the hilum. R—right, L—left, S—superior, I—inferior.

The kidney and its vascular structures were identified in all examined sides (400/400, 100%). Interobserver agreement was excellent, with a Cohen’s kappa coefficient (κ) of 0.892. Anatomical variations predominated, accounting for 67.4% of cases (269/400), whereas the classical vein–artery–pelvis (VAP) configuration was observed in only 32.6% of sides (131/400) (Figure 1).

**Figure 2:**
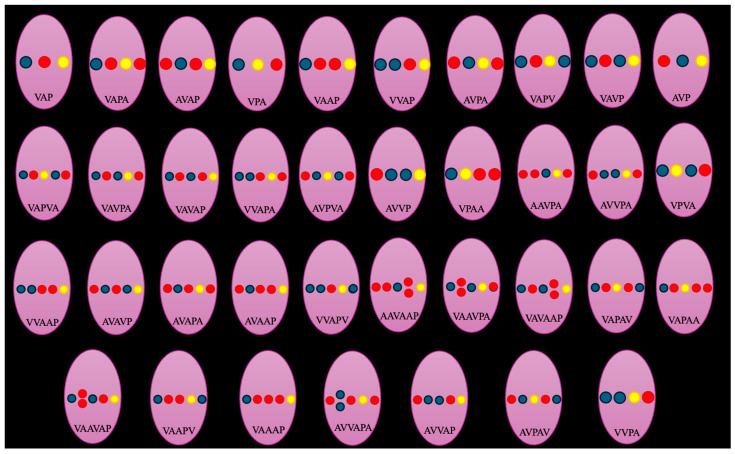
Schematic illustration of the 37 variant patterns of renal hilar architecture. The renal hilum is depicted in purple, the vein in blue, the artery in red, and the pelvis in yellow. Each pattern represents the anterior-to-posterior arrangement of hilar structures as observed on multiplanar CT reconstructions.

The observed morphological variability was categorized into 37 distinct hilar sequence patterns, as summarized in Table 1 and Figure 2.

**Figure 3:**
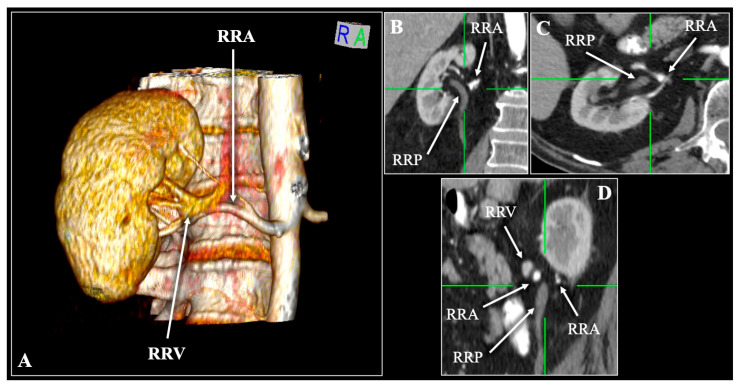
A contrast-enhanced computed tomography (CT) scan of a 64-year-old male patient illustrating the most common vascular variation: Vein (V)—Artery (A)—Pelvis (P)—Artery (A). The image includes a three-dimensional reconstruction (**A**) and multiplanar reconstructions in coronal (**B**), axial (**C**), and sagittal (**D**) planes. Notably, there is a single right renal vein (RRV) and a bifurcated right renal artery (RRA) entering the hilum. Additionally, the renal pelvis (RRP) is observed exiting between the two arteries on the sagittal reconstruction depicted in Figure 3D. R—right, L—left, S—superior, I—inferior.

**Figure 4:**
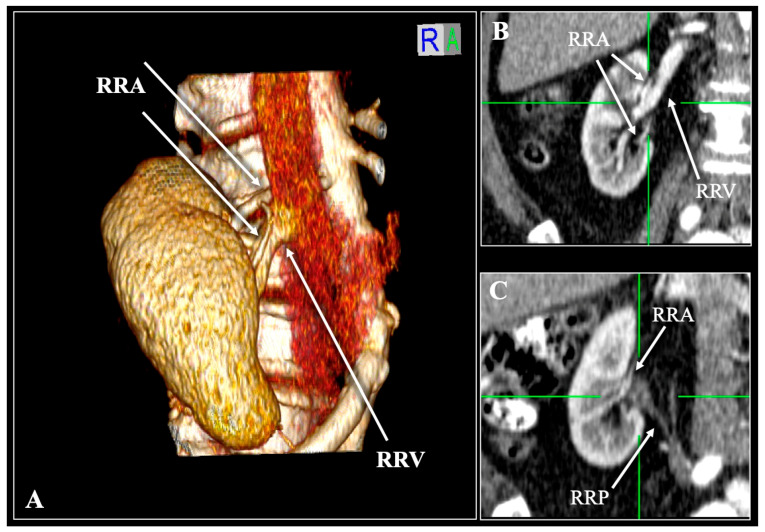
Contrast-enhanced computed tomography (CT) scan of a 58-year-old female patient demonstrating the Artery (A)—Vein (V)—Artery (A)—Pelvis (P) variation. Three-dimensional reconstruction (**A**) and multiplanar reconstruction with coronal (**B**,**C**) slices. A single right renal vein (RRV) and a bifurcated right renal artery (RRA) entering the hilum. RRP—right renal pelvis. R—right, L—left, S—superior, I—inferior.

**Figure 5:**
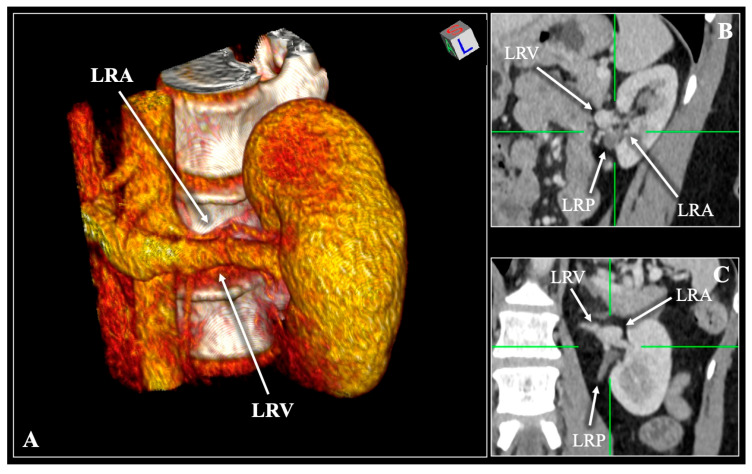
Contrast-enhanced computed tomography (CT) scan of a 57-year-old female patient demonstrating the Vein (V)—Pelvis (P)—Artery (A). Three-dimensional reconstruction (**A**) and multiplanar reconstruction with sagittal (**B**) and coronal (**C**) slices. Note the renal pelvis (LRP) interfering between the left renal vein (LRV) and artery (LRA) in sagittal and coronal reconstruction (**B**,**C**). R—right, L—left, S—superior, I—inferior.

**Figure 6:**
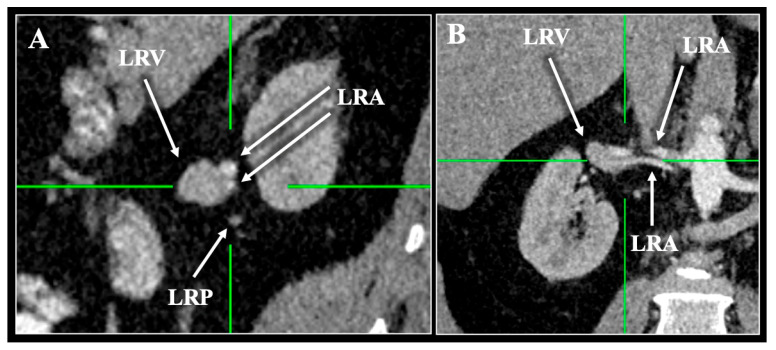
Contrast-enhanced computed tomography (CT) scan of a 48-year-old male patient demonstrating the Vein (V)—Artery (A)—Artery (A)—Pelvis (P) variation. Multiplanar reconstruction with sagittal (**A**) and coronal (**B**) slices. Two left renal arteries (LRAs) and a single renal vein (LRV) enter and exit the hilum. LRP—left renal pelvis. R—right, L—left, S—superior, I—inferior.

**Figure 7:**
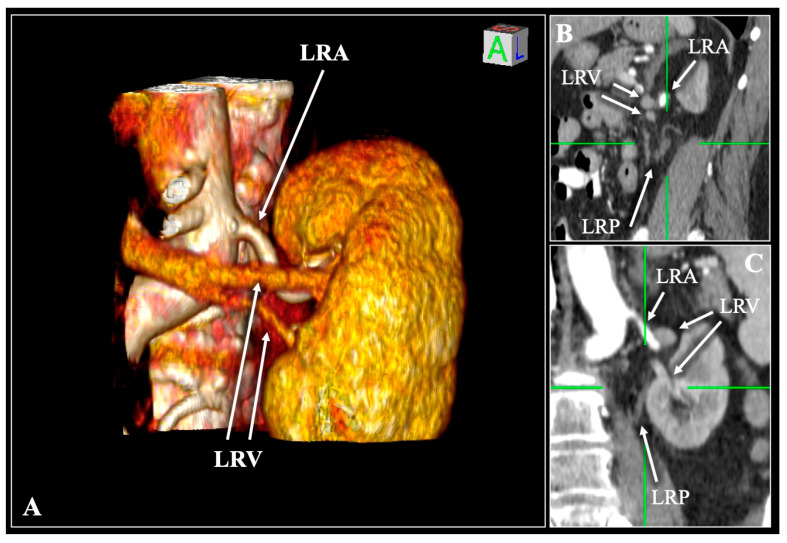
Contrast-enhanced computed tomography (CT) scan of a 70-year-old female patient demonstrating the Vein (V)—Vein (V)—Artery (A)—Pelvis (P). Three-dimensional reconstruction (**A**) and multiplanar reconstruction with sagittal (**B**) and coronal (**C**) slices. Two left renal veins (LRV) and a single left renal artery (LRA) enter and exit the hilum. LRP—left renal pelvis. R—right, L—left, S—superior, I—inferior.

The prevalence of the classical VAP sequence differed significantly between sexes [*p* = 0.046, OR (male) = 0.64], with females more frequently exhibiting this configuration than males (37.7% vs. 27.9%, respectively) (Table 1). In contrast, no significant difference was observed with respect to laterality (*p* = 0.887), as the VAP pattern occurred in 32.0% of left sides and 33.2% of right sides (Table 1). The five most common variant sequences were VAPA (9%), AVAP (7.8%), VPA (7%), VAAP (7%), and VVAP (6%) (Figure 3, Figure 4, Figure 5, Figure 6 and Figure 7).

**Figure 8:**
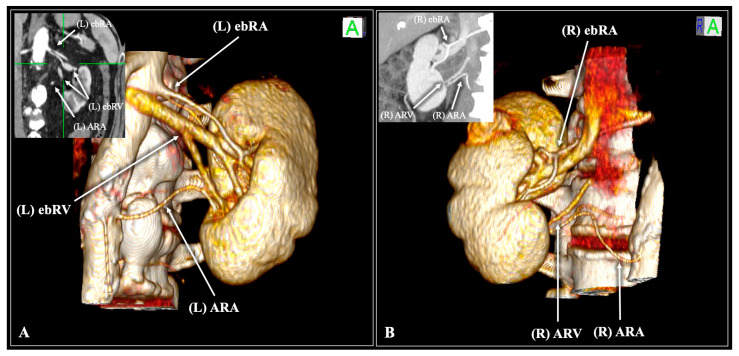
Contrast-enhanced computed tomography (CT) scan of a 61-year-old male (**A**) and a 68-year-old female patient (**B**) with three-dimensional and coronal reconstruction. The male patient (**A**) has an early bifurcated renal artery (ebRA) and vein (ebRV), as well as an accessory renal artery (ARA). The female patient (**B**) has an ebRA, an ARA, and an accessory renal vein (ARV). Note that the male patient (A) is demonstrating Artery (A)—Vein (V)—Vein (V)—Artery (A)—Pelvis (P)—Artery (A) variant. R—right, L—left, S—superior, I—inferior.

A typical RA configuration was observed in 56.8% of cases (227/400). Arterial variations were common, with ebRAs identified in 30.5% (122/400) and ARAs in 10.8% (43/400) (Figure 8; Table 2).

Significant sexual dimorphism in arterial morphology was observed [*p* < 0.001, OR (male) = 2.37], with males exhibiting a higher prevalence of arterial variants than females (53.4% vs. 32.3%, respectively) (Table 2). Additionally, laterality was significantly associated with arterial branching patterns (*p* = 0.026), with ebRAs more frequently observed on the right side compared to the left (37.0% vs. 24.0%, respectively).

Typical venous anatomy was observed in 73.8% of cases (295/400). The most frequent venous variants were ebRVs, identified in 18.0% (72/400) of cases, and ARVs, present in 8.0% (32/400) of cases (Figure 8; Table 3). No significant sex-based differences were observed in venous variation patterns [*p* = 0.249, OR (male) = 1.32]. However, laterality demonstrated a significant association (*p* = 0.002), with ARVs more commonly identified on the right side compared to the left (12.5% vs. 3.5%, respectively), whereas ebRVs were more prevalent on the left (30.0% vs. 6.0%, respectively) (Table 3).

## 4. Discussion

This radioanatomical study demonstrates that variability of the renal hilum is not an exception but rather a defining characteristic of renal architecture. The predominance of variant patterns, observed in 67.4% of examined sides, together with the identification of 37 distinct hilar sequence configurations, fundamentally challenges the classical assumption that the VAP sequence represents the normative arrangement. Instead, the VAP configuration should be regarded as one of several common patterns rather than the anatomical standard. The extent and diversity of this variability carry direct and clinically relevant implications across the spectrum of retroperitoneal surgical procedures, particularly in contexts requiring precise hilar dissection and vascular control.

Herein, we are proposing a simplified classification system of these 37 variants identified in the current study:

1. Simple configuration—typical pattern (VAP) or minor deviations (such as VPA)- with the presence of a single renal artery and vein. This type was identified on 38.8% of sides (155 sides).
2. Intermediate configuration—modified sequence (such as AVAP, VAAP, VAPA) with the presence of variation from the artery or the vein (single-system variant). This type was observed on 47.2% of sides (189).
3. Complex configuration—multi-layered or multiple vessel sequence with the presence of variation from both artery and vein. This type was recorded on 14.0% of sides (56 sides).

### 4.1. The Kidney Hilar Variation (Figure 9)

The identification of the VAP sequence in only 32.6% of examined sides is markedly lower than the prevalence implied by classical anatomical teaching. The existing literature on hilar sequence classification remains limited, with prior studies typically constrained by small sample sizes, reliance on cadaveric material, or a narrow focus on isolated vascular variants rather than comprehensive sequence characterization [2,11]. In contrast, the present study provides a systematic, population-level evaluation of composite hilar architecture.

The five most prevalent variant sequences—VAPA (9.0%), AVAP (7.8%), VPA (7.0%), VAAP (7.0%), and VVAP (6.0%)—collectively account for 36.8% of all examined sides; however, no single configuration predominates. This distribution underscores the absence of a dominant anatomical pattern and highlights the inherently heterogeneous nature of renal hilar organization.

The clinical significance of variations in hilar sequence manifests at various levels of surgical decision-making. In the AVAP pattern—present in 7.8% of cases—the artery is positioned anteriorly, thereby reversing the anticipated dissection order and increasing the risk of inadvertent arterial injury during initial identification of the vein [12]. Likewise, the VPA configuration, in which the renal pelvis is situated immediately posterior to the vein without an interposed artery, may be erroneously interpreted during laparoscopic dissection as an entirely exposed hilum, whereas the arterial component remains unrecognized and undissected [11,12].

These anatomical challenges are further intensified in robot-assisted retroperitoneal procedures, where the limited operative field and lack of tactile feedback demand meticulous preoperative anatomical comprehension. In such circumstances, dependence on a precise three-dimensional mental reconstruction of hilar anatomy—obtained from preoperative imaging—is crucial to prevent critical misidentification and intraoperative complications [2,12].

**Figure 9:**
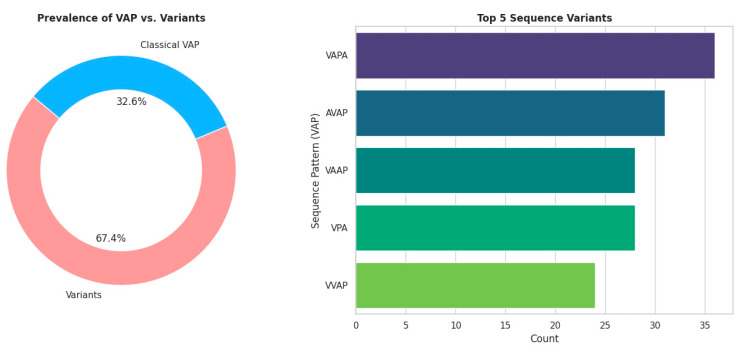
Graphical representation of the prevalence and distribution of renal hilar sequence patterns. (**Left**) Donut chart illustrating the proportion of the classical vein–artery–pelvis (VAP) configuration compared to anatomical variants. (**Right**) Horizontal bar chart demonstrating the frequency of the five most common sequence variants (VAPA, AVAP, VAAP, VPA, and VVAP). Values are expressed as absolute counts and percentages of total renal units (*n* = 400).

### 4.2. Sexual Dimorphism and Laterality on Hilar Architecture

A noteworthy sex-related disparity was identified in the prevalence of the classical VAP sequence (*p* = 0.046), with females exhibiting the typical configuration more frequently than males (37.7% vs. 27.9%). This suggests that males are disproportionately represented among variant patterns, a finding of clinical significance given the well-documented male predominance in renal cell carcinoma [3]. Consequently, the population most commonly subjected to renal surgery is also less likely to display standard hilar anatomy, thereby elevating the potential for intraoperative anatomical complexity. This sex-based effect is further reinforced by the marked sexual dimorphism in arterial morphology (*p* < 0.001), suggesting that greater arterial complexity in males may contribute to the higher prevalence of non-classical hilar configurations.

In contrast, laterality did not significantly influence the prevalence of the VAP pattern (*p* = 0.887), with similar distributions observed between left and right sides (32.0% vs. 33.2%, respectively). This near-symmetrical distribution indicates that overall hilar variability is consistently expressed bilaterally, despite specific arterial and venous variant subtypes exhibiting side-dependent differences.

### 4.3. Variability of Renal Vessels

The ebRAs represented the most prevalent arterial variant in the present cohort (30.5%), with a significant right-sided predominance (37.0% vs. 24.0%, *p* = 0.026). The longer extrahilar course of the right RA (RRA)—which traverses the retroperitoneum posterior to the inferior vena cava (IVC) before reaching the kidney—provides a greater anatomical length within which premature division can occur, thereby explaining this laterality. The distinction between ebRAs and ARAs is of critical importance for surgical planning [5]. Unlike ARAs, ebRAs do not represent independent aortic origins; rather, their clinical significance lies in the fact that arterial division occurs proximal to the hilum, rendering standard single-clamp control of the main renal artery unreliable or unfeasible [13].

In the context of partial nephrectomy, preoperative identification of ebRAs is essential for planning selective arterial clamping, whereby only the segmental branch supplying the tumor-bearing territory is occluded to minimize global warm ischemia. The success of this strategy depends on accurate preoperative CT delineation of branching patterns and their correspondence with renal segmental anatomy [14,15]. In living-donor nephrectomy, the presence of a right-sided ebRA further compounds the inherent technical challenges of the right kidney, which is already further constrained by the anatomical limitations of a shorter RV by a shorter RV and more limited operative length for vascular control [16,17].

ARAs were identified in 10.8% of examined sides, consistent with the lower end of the previously reported prevalence range (8–30%) across different populations and methodologies [2,4]. As embryological remnants of the lateral splanchnic branches, ARAs function as end-arteries supplying discrete renal segments, and their clinical relevance derives directly from this segmental perfusion pattern. Inadvertent ligation of an unrecognized ARA may result in segmental infarction, potentially leading to ischemic complications or anastomotic failure in transplant recipients [18,19].

**Figure 10:**
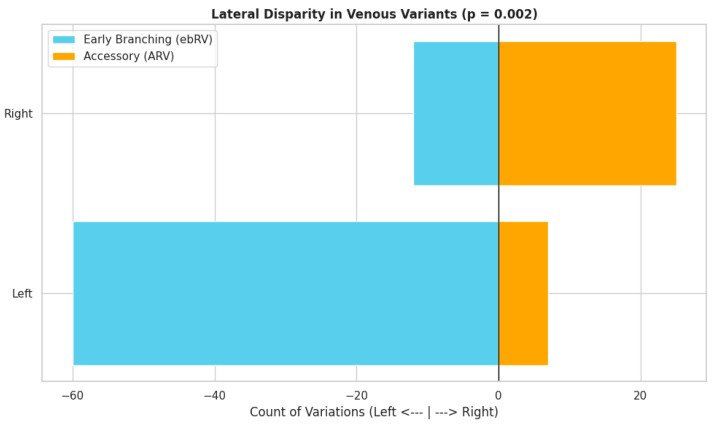
Mirror bar chart illustrating lateral disparities in renal venous variations. Early-branching renal veins (ebRVs) and accessory renal veins (ARVs) are displayed by laterality. Left-sided counts are presented as negative values and right-sided counts as positive values for visual comparison. A significant association between laterality and venous variation type was observed (*p* = 0.002). ebRVs were more prevalent on the left side, whereas ARVs predominated on the right.

Venous variations demonstrated a distinct laterality pattern (Figure 10). ARVs were predominantly right-sided (12.5% vs. 3.5%), reflecting persistent embryological tributaries to the IVC that fail to consolidate into a single venous trunk [6]. In contrast, ebRVs were significantly more prevalent on the left (30.0% vs. 6.0%), a finding attributable to the greater length and anastomotic complexity of the left renal vein, which provides increased opportunity for intrahilar division prior to confluence [6].

These laterality-dependent patterns have direct surgical implications. During laparoscopic left donor nephrectomy, the presence of an ebRV implies that nearly one-third of harvested kidneys may exhibit venous division at the hilum. Failure to recognize early venous branching can result in incomplete dissection, retained tributaries, or the need for complex bench reconstruction [6,20]. Conversely, in right-sided procedures—including radical nephrectomy, donor procurement, and IVC-adjacent dissections—the presence of ARVs necessitates careful inspection of the caval junction, as unrecognized veins may be inadvertently transected during renal mobilization, leading to significant hemorrhage [21].

### 4.4. Embryological Basis of Variants

An understanding of the embryological basis of renal vascular variation is essential for interpreting the prevalence and distribution of variants observed in the present study.

The definitive renal vasculature arises from the lateral splanchnic branches of the dorsal aorta, which supply the metanephros during its ascent from the sacral to the lumbar retroperitoneum between the sixth and ninth weeks of gestation [22,23]. Under normal conditions, the caudal arterial branches regress sequentially as more cranial vessels assume dominance, resulting in the persistence of a single renal artery at the L1–L2 level [22,23]. Incomplete regression of these transient vessels leads to the persistence of ARAs, which should therefore be regarded as embryological remnants rather than true anomalies [4,24]. The male-predominant distribution of arterial variants observed in this study may reflect sex-specific differences in vascular remodeling during nephrogenesis, potentially mediated by hormonal or transcriptional influences [25].

The ebRAs arise through a distinct but related mechanism. Rather than representing persistence of independent aortic branches, ebRAs reflect premature division of the definitive renal artery prior to hilar entry [5,26]. This pattern likely results from the incorporation of segmental branching points into the main arterial trunk during vascular development, effectively relocating the hilar bifurcation to an extrahilar position [26]. The right-sided predominance of ebRAs identified in this series is anatomically consistent, as the right renal artery follows a longer retroperitoneal course, passing posterior to the inferior vena cava before reaching the hilum. This extended extrahilar trajectory may increase the likelihood of premature branching before hilar entry [27].

Renal venous variants arise from the complex remodeling of the embryological venous plexus. The renal veins develop from the subcardinal venous system, which undergoes asymmetric regression and reorganization [22,23]. The left renal vein, formed through anastomosis with the right-sided inferior vena cava precursor, acquires a longer course and more complex anastomotic pattern than the right [22,23]. ARVs, particularly prevalent on the right side, represent persistent embryonic venous channels that fail to consolidate into a single renal vein [2,6,28]. This embryological asymmetry provides a mechanistic explanation for the side-dependent distribution of venous variants observed in the present study.

### 4.5. Clinical and Surgical Consideration of Renal Hilar Architecture

The implications of the present study are most directly applicable to retroperitoneal surgical approaches, in which the renal hilum is the first critical anatomical landmark encountered. The integration of our proposed classification into routine preoperative CT reporting provides a concise and operationally meaningful schema. Instead of listing individual vessels, a radiologist can categorize a patient as simple/intermediate/complex—alerting the surgeon to the need for 3D reconstruction and potential selective clamping. This structured approach would significantly enhance communication and improve operative outcomes [29].

The retroperitoneal laparoscopic approach, increasingly favored for its direct access to the renal pedicle without bowel mobilization, requires early and accurate identification of the RA before venous or ureteral dissection [30]. In configurations in which the artery is anterior or demonstrates early branching, the expected arterial plane may be absent or displaced, necessitating modification of the dissection strategy. Accordingly, preoperative characterization of hilar sequence patterns using CT imaging directly informs the initial surgical trajectory and reduces the risk of intraoperative misidentification [30].

Robot-assisted partial nephrectomy further underscores the importance of precise anatomical mapping, particularly in the context of selective or zero-ischemia techniques aimed at maximizing renal function preservation. These approaches are critically dependent on accurate delineation of segmental arterial anatomy, which is directly influenced by hilar configuration and branching patterns [31].

Renal transplantation represents the clinical setting in which detailed preoperative characterization of hilar anatomy has the greatest immediate impact. The choice between left and right kidney procurement in living donors is influenced by multiple anatomical variables, and the present findings provide a quantitative framework for risk stratification. The observed distribution of vascular variants—specifically, the higher prevalence of ebRAs on the right (37.0%), ebRVs on the left (30.0%), and ARVs on the right (12.5%)—demonstrates that neither kidney is anatomically straightforward.

For left-sided donor nephrectomy, which is generally preferred due to the longer RV, the substantial prevalence of ebRVs necessitates systematic preoperative venous mapping, and the surgical technique must ensure that venous division is performed at or beyond the point of confluence [16,17,18,20]. Conversely, for right-sided procurement, the combination of a shorter RV and a higher incidence of early arterial branching introduces additional technical complexity that must be carefully considered during donor selection. In deceased-donor transplantation, where intraoperative assessment is limited by time constraints, systematic preoperative evaluation of CT using a structured hilar classification framework enables anticipation of vascular complexity and facilitates operative planning [16,17,18,20].

A key contribution of the present study is the demonstration that contrast-enhanced CT, combined with multiplanar and three-dimensional reconstruction, can reliably delineate the full complexity of renal hilar architecture with sufficient resolution to guide surgical decision-making [27]. The integration of hilar sequence classification (V–A–P patterns) with standardized vascular variant taxonomy (ARA, ebRA, ARV, ebRV) provides a concise and clinically actionable reporting framework. The adoption of a structured radiological reporting template incorporating sequence classification, variant identification, and laterality would significantly enhance communication between radiologists and surgeons, thereby improving operative safety and outcomes [29].

### 4.6. Limitations and Future Directions

Several limitations of the present study should be acknowledged. First, the retrospective single-institution design and the use of a single-center cohort may limit the generalizability of the findings to populations with different ethnic or anthropometric characteristics and may introduce sampling bias, as the study population may not fully represent the anatomical diversity of the general population. Second, classification was based on radiological assessment using multiplanar and three-dimensional CT reconstructions rather than direct anatomical dissection. Although high-resolution imaging was employed, the possibility of misclassification in cases with complex or overlapping structures cannot be entirely excluded. Additionally, the lack of clinical or intraoperative correlation limits the ability to directly assess the surgical relevance of the observed anatomical patterns. Finally, the proposed three-tier classification system remains unvalidated.

Future research should concentrate on the prospective validation of the proposed radiological framework. It is necessary to conduct studies that correlate preoperative CT-based hilar classification with intraoperative findings to establish its predictive accuracy and clinical reliability. Furthermore, integrating functional imaging parameters, such as split renal function assessment, may further enhance the clinical applicability of anatomical characterization. Multicenter studies with larger and more diverse populations would further strengthen the external validity of the proposed classification system.

## 5. Conclusions

The current study illustrates that variability in renal hilar architecture is commonplace rather than exceptional, with the classical VAP sequence observed in merely 32.6% of the examined sides, accompanied by 37 distinct hilar configurations. Therefore, we propose a three-tier classification system (simple, intermediate, and complex) to support preoperative anatomical assessment in retroperitoneal surgery. Anatomical variation was significantly more prevalent in males and demonstrated distinct laterality-dependent patterns for both arterial and venous variants, thereby further emphasizing the complexity of renal hilar organization. In conclusion, given that classical hilar architecture is the exception rather than the rule, the adoption of a systematic imaging-based classification may improve surgical planning and intraoperative orientation in modern retroperitoneal procedures. These findings challenge longstanding anatomical assumptions that continue to influence surgical practice. To enhance precision, preserve parenchyma, and enable individualized planning, a thorough imaging-based understanding of hilar anatomical variability is essential to reduce complications and maximize surgical outcomes.

## Figures and Tables

**Table 1:** Summary of the results of the current study regarding the sequence pattern. V—vein, A—artery, P—pelvis.

Sequence Variant	Total Cases (%)	Left (%)	Right (%)	Female (%)	Male (%)
VAP	131 (32.6%)	64 (32.0%)	66 (33.2%)	72 (37.7%)	58 (27.9%)
VAPA	36 (9.0%)	11 (5.5%)	25 (12.6%)	16 (8.4%)	20 (9.6%)
AVAP	31 (7.8%)	8 (4.0%)	23 (11.6%)	13 (6.8%)	18 (8.7%)
VPA	28 (7.0%)	12 (6.0%)	16 (8.0%)	19 (9.9%)	9 (4.3%)
VAAP	28 (7.0%)	15 (7.5%)	13 (6.5%)	13 (6.8%)	15 (7.2%)
VVAP	24 (6.0%)	14 (7.0%)	10 (5.0%)	18 (9.4%)	6 (2.9%)
AVPA	18 (4.5%)	9 (4.5%)	9 (4.5%)	4 (2.1%)	14 (6.7%)
VAPV	16 (4.0%)	12 (6.0%)	4 (2.0%)	8 (4.2%)	8 (3.8%)
VAVP	12 (3.0%)	11 (5.5%)	1 (0.5%)	3 (1.6%)	9 (4.3%)
AVP	11 (2.8%)	9 (4.5%)	2 (1.0%)	7 (3.7%)	4 (1.9%)
VAPVA	10 (2.5%)	7 (3.5%)	3 (1.5%)	2 (1.0%)	8 (3.8%)
VAVPA	8 (2.0%)	6 (3.0%)	2 (1.0%)	3 (1.6%)	5 (2.4%)
VAVAP	7 (1.8%)	2 (1.0%)	5 (2.5%)	1 (0.5%)	6 (2.9%)
VVAPA	4 (1.0%)	1 (0.5%)	3 (1.5%)	1 (0.5%)	3 (1.4%)
AVPVA	3 (0.8%)	2 (1.0%)	1 (0.5%)	1 (0.5%)	2 (1.0%)
AVVP	3 (0.8%)	1 (0.5%)	2 (1.0%)	2 (1.0%)	1 (0.5%)
VPAA	3 (0.8%)	2 (1.0%)	1 (0.5%)	0 (0.0%)	3 (1.4%)
AAVPA	2 (0.5%)	2 (1.0%)	0 (0.0%)	1 (0.5%)	1 (0.5%)
AVVPA	2 (0.5%)	0 (0.0%)	2 (1.0%)	0 (0.0%)	2 (1.0%)
VPVA	2 (0.5%)	2 (1.0%)	0 (0.0%)	0 (0.0%)	2 (1.0%)
VVAAP	2 (0.5%)	0 (0.0%)	2 (1.0%)	1 (0.5%)	1 (0.5%)
AVAVP	2 (0.5%)	1 (0.5%)	1 (0.5%)	1 (0.5%)	1 (0.5%)
AVAPA	2 (0.5%)	1 (0.5%)	1 (0.5%)	1 (0.5%)	1 (0.5%)
AVAAP	2 (0.5%)	0 (0.0%)	2 (1.0%)	0 (0.0%)	2 (1.0%)
VVAPV	1 (0.3%)	1 (0.5%)	0 (0.0%)	1 (0.5%)	0 (0.0%)
AAVAAP	1 (0.3%)	0 (0.0%)	1 (0.5%)	0 (0.0%)	1 (0.5%)
VAAVPA	1 (0.3%)	1 (0.5%)	0 (0.0%)	0 (0.0%)	1 (0.5%)
VAVAAP	1 (0.3%)	1 (0.5%)	0 (0.0%)	0 (0.0%)	1 (0.5%)
VAPAV	1 (0.3%)	1 (0.5%)	0 (0.0%)	1 (0.5%)	0 (0.0%)
VAPAA	1 (0.3%)	1 (0.5%)	0 (0.0%)	0 (0.0%)	1 (0.5%)
VAAVAP	1 (0.3%)	0 (0.0%)	1 (0.5%)	1 (0.5%)	0 (0.0%)
VAAPV	1 (0.3%)	1 (0.5%)	0 (0.0%)	1 (0.5%)	0 (0.0%)
VAAAP	1 (0.3%)	0 (0.0%)	1 (0.5%)	0 (0.0%)	1 (0.5%)
AVVAPA	1 (0.3%)	1 (0.5%)	0 (0.0%)	0 (0.0%)	1 (0.5%)
AVVAP	1 (0.3%)	1 (0.5%)	0 (0.0%)	0 (0.0%)	1 (0.5%)
AVPAV	1 (0.3%)	0 (0.0%)	1 (0.5%)	0 (0.0%)	1 (0.5%)
VVPA	1 (0.3%)	0 (0.0%)	1 (0.5%)	0 (0.0%)	1 (0.5%)

**Table 2:** Summary of the results of the current study regarding the arterial variants.

Variation Type	Total Cases (%)	Left (%)	Right (%)	Female (%)	Male (%)
Normal	227 (56.8%)	125 (62.5%)	102 (51.0%)	130 (67.7%)	97 (46.6%)
Early Branching	122 (30.5%)	48 (24.0%)	74 (37.0%)	50 (26.0%)	72 (34.6%)
Accessory	43 (10.8%)	23 (11.5%)	20 (10.0%)	12 (6.2%)	31 (14.9%)
Accessory + Early Branching	8 (2.0%)	4 (2.0%)	4 (2.0%)	0 (0.0%)	8 (3.8%)

**Table 3:** Summary of the results of the current study regarding the venous variants.

Variation Type	Total Cases (%)	Left (%)	Right (%)	Female (%)	Male (%)
Normal	295 (73.8%)	133 (66.5%)	162 (81.0%)	147 (76.6%)	148 (71.2%)
Early Branching	72 (18.0%)	60 (30.0%)	12 (6.0%)	32 (16.7%)	40 (19.2%)
Accessory	32 (8.0%)	7 (3.5%)	25 (12.5%)	12 (6.2%)	20 (9.6%)
Accessory + Early Branching	1 (0.2%)	0 (0.0%)	1 (0.5%)	1 (0.5%)	0 (0.0%)

## Data Availability

Please contact the authors for data requests.

## Abbreviations

The following abbreviations are used in this manuscript:

CT	Computed Tomography
3D	Three-dimensional
CI	Confidence Interval
SD	Standard Deviation
V	Vein
A	Artery
P	Pelvis
VAP	Vein–Artery–Pelvis (Classical sequence)
RA	Renal Artery
RV	Renal Vein
ebRA	Early branching Renal Artery
ARA	Accessory Renal Artery
ebRV	Early branching Renal Vein
ARV	Accessory Renal Vein
RRA	Right Renal Artery
LRA	Left Renal Artery
RRV	Right Renal Vein
LRV	Left Renal Vein
RRP	Right Renal Pelvis
LRP	Left Renal Pelvis
IVC	Inferior Vena Cava
GEE	Generalized Estimating Equations
HU	Hounsfield Units
